# The mitochondrial genome of one ‘twisted-wing parasite’ *Xenos* cf. *moutoni* (Insecta, Strepsiptera, Xenidae) from Gaoligong Mountains, Southwest of China

**DOI:** 10.1080/23802359.2021.1872443

**Published:** 2021-02-11

**Authors:** Ru Zhang, Jun Li, Chuyang Mao, Zhiwei Dong, Jinwu He, Guichun Liu, Ruoping Zhao, Wen Wang, Xueyan Li

**Affiliations:** a School of Ecology and Environment, Northwestern Polytechnical University, Xi’an, Shaanxi, China; b State Key Laboratory of Genetic Resources and Evolution, Kunming Institute of Zoology, Chinese Academy of Sciences, Kunming, Yunnan, China; c Center for Excellence in Animal Evolution and Genetics, Kunming, Yunnan, China

**Keywords:** Mitogenome, Strepsiptera, *Xenos* cf. *moutoni*, Southwest of China, phylogenetic analysis

## Abstract

The nearly complete mitochondrial genome (mitogenome) of *Xenos* cf. *moutoni*, one twisted-wing parasite on wasp *Vespa velutina* from Southwest of China, is described in this study. The total length of this mitogenome is 16,717 bp, containing 13 protein-coding genes (PCGs), 22 transfer RNA genes (tRNAs), two ribosomal RNA genes (rRNAs) and an incomplete A + T-rich control region . All of the 13 PCGs are initiated with canonical ATN (N represents A, T, G, C) as start codons; 8 PCGs are terminated with a complete typical stop codon TAA, and the remaining five PCGs (*cox2*, *cox3*, *nad3*, *nad4* and *nad5*) have an incomplete stop codon with just a T. The phylogenetic analysis based on the nucleotide sequences of PCGs and rRNAs indicates that *Xenos* cf*. moutoni* has a close relationship with *Xenos vesparum*, confirming its placement in the family Xenidae.

Strepsiptera, often known as ‘twisted wing’ parasitoids, is a kind of enigmatic insects with a complex life cycle and unusual morphology (Pohl and Beutel 2008; Cook 2014; Kathirithamby 2018). It is a small order of holometabolous insects, comprising 630 species in 15 families, five of which are extinct (Kathirithamby 2018). All strepsipterans utilize insects as hosts, and their host associations are found in seven insect orders (Cook 2014). Among 10 extant families, the family Mengenillidae use apterygote insect (Zygentoma) as host, and the families, Stylopidae and Xenidae, are known to use aculeate Hymenoptera as hosts: Stylopidae parasitizes bees (Apidae), and Xenidae parasitizes wasps (Crabronidae, Sphecidae, and Vespidae) (Pohl and Beutel 2008; Cook 2014). The Xenidae includes *ca*. 110 described species (*ca*. 19%) and thus is one of the species-rich strepsipteran families (Pohl and Beutel 2008). To date, only three mitogenomic sequences (*Mengenilla australiensis* and *M. moldrzyki* in Mengenillidae and *Xenos vesparum* in Xenidae) were reported (Carapelli et al. 2006; McMahon et al. 2009; Niehuis et al. 2012).

Twisted-wing insect species *Xenos* cf. *moutoni* (males and famales) were collected from the body of wasps (Hymenoptera, Vespidae, *Vespa velutina*) in one nest was collected from Gaoligong Mountains, Xiangda Township, Longling County, Yunnan Province (98°43′26″E, 24°26′38″N), China, on 20 December 2019 by local villagers. With reference to male morphological traits of strepsipteran insects (Buysson 1903; Kifune and Maeta 1985; Kathirithamby and Engel 2014) and the published strepsipteran mitochondrial *cox1* data (Benda et al. 2019), this species is identified as *Xenos* cf. *moutoni* in the family Xenidae. The voucher specimens (voucher no. KIZ0127514) are stored in Kunming Natural History Museum of Zoology, Chinese Academy of Sciences. Total genomic DNA (gDNA) of one female adult was isolated using a Gentra Puregene Blood kit (Qiagen, Hilden, Germany) based on instructions. Library (150-bp insert size) was prepared and sequenced on Illumina Novaseq 6000 (Novogene, Beijing, China). The 126,444,619 clean reads obtained were used to execute the assembly using both MitoZ v2.3 (Meng et al. 2019) and NOVOPlasty v4.2 (Dierckxsens et al. 2017). Gene annotation was performed using the MITOS2 webserver (Bernt et al. 2013) with manual correction.

Both assembled methods (MitoZ and NOVOPlasty) produced the same sequence except A + T-rich region which is longer in NOVOPlasty assembly than in MitoZ assembly. The mitogenome of *Xenos* cf. *moutoni* (GenBank accession No. MW222190) using NOVOPlasty was 16,717 bp in length and 1,658,750 reads were mapped to the mitogenome with 6,904 mean coverage. The linear mitogenome was considered to be incomplete because its two terminuses lack repetitive overlaps with longer than 25 bp (Tang et al. 2014). It contained 13 protein-coding genes (PCGs), 22 transfer RNA genes (tRNAs), two ribosomal RNA genes (rRNAs) and an incomplete control (A + T-rich) region (about 2107 bp). All 13 PCGs are 10,676 bp in length which contain 35.59% A, 9.49% G, 44.30% T and 10.62% C with a strong bias toward A + T (79.89%). All of 13 PCGs use ‘ATN’ (N represents A, T, G, C) as the start codons; eight of them are terminated with typical stop codon ‘TAA’, and the other five (*cox2*, *cox3*, *nad3*, *nad4* and *nad5*) have an incomplete stop codon ‘T––’. The length of 22 tRNAs ranges from 56 bp to 69 bp and the size of small rRNA (*s-rRNA*) and large rRNA (*l-rRNA*) is 735 bp and 1234 bp, respectively. The arrangement of all 13 PCGs in *Xenos* cf. *moutoni* is consistent with those of two other strepsipterans (*M. australiensis* and *X. vesparum*) and the luminous beetle *R. lufengensis* (Carapelli et al. 2006; Li et al. 2007; McMahon et al. 2009). However, some tRNA gene order changes are observed among the four species in those regions between *nad3* and *nad5*, *nad5* and *nad4* as well as *s-rRNA* and *nad2* and in the location of the *trnL1* (Carapelli et al. 2006; Li et al. 2007; McMahon et al. 2009).

Phylogenetic interference was performed among *Xenos* cf. *moutoni* and other three reported strepsiperan species (Carapelli et al. 2006; McMahon et al. 2009; Niehuis et al. 2012) with *Tribolium castaneum* (Coleopetera, Tenebrionidae) as an outgroup. The 13 PCGs and two rRNAs from these species were aligned and concatenated using MEGA-X (Kumar et al. 2018) and then trimmed the unreliably regions using trimAl v1.4 (gt = 0.5) (Capella-Gutierrez and Silla-Martinez 2009). The phylogenetic tree was constructed based on the concatenated nucleotide sequence using RAxML v8.2.10 (Stamatakis 2014) with the GTR + G + I model. Our result indicates that *Xenos* cf. *moutoni* has a close relationship with *X. vesparum* in the family Xenidae with high bootstrap values (Figure 1), confirming its placement in the family Xenidae.

**Figure 1.**
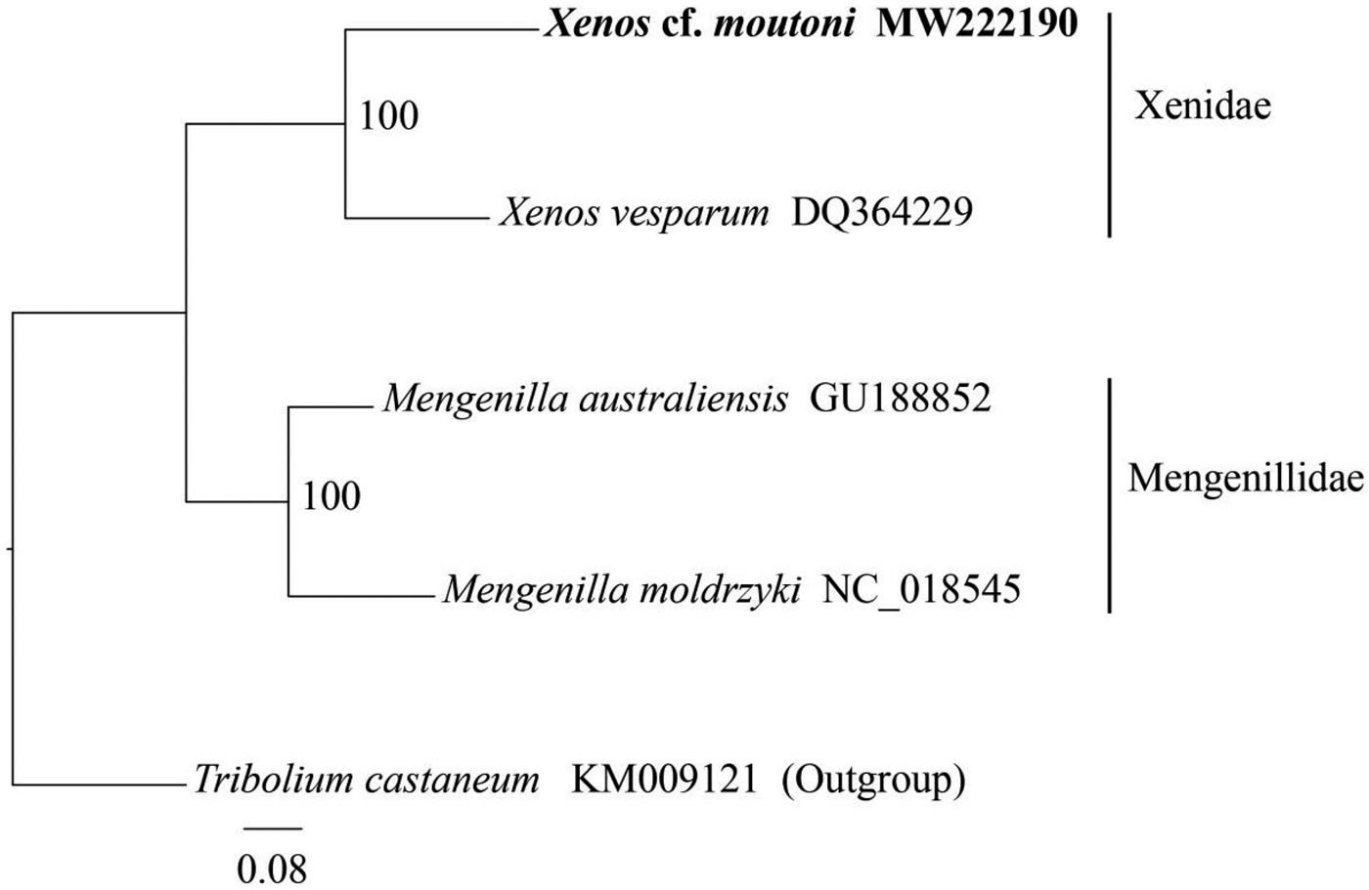
The phylogenetic tree of four strepsipteran species based on the nucleotide sequence of concatenated 13 protein-coding genes (PCGs) and two ribosomal RNA genes (rRNAs) using maximum likelihood (ML) analysis. Red flour beetle *Tribolium castaneum* was selected as the outgroup. The values at the nodes show the bootstrap support calculated using the maximum likelihood method with 100 replicates.

## Data Availability

The genome sequence data that support the findings of this study are openly available in GenBank of NCBI at https://www.ncbi.nlm.nih.gov, under the accession no. MW222190. The associated BioProject, Bio-Sample and SRA numbers are PRJNA681068, SAMN16898475 and SRR13154212, respectively.

## References

[CIT0001] Benda D, Nakase Y, Straka J. 2019. Frozen Antarctic path for dispersal initiated parallel host-parasite evolution on different continents. Mol Phylogenet Evol. 135:67–77. 30849429 10.1016/j.ympev.2019.02.023

[CIT0002] Bernt M, Donath A, Juhling F, Externbrink F, Florentz C, Fritzsch G, Putz J, Middendorf M, Stadler PF. 2013. MITOS: improved de novo metazoan mitochondrial genome annotation. Mol Phylogenet Evol. 69(2):313–319. 22982435 10.1016/j.ympev.2012.08.023

[CIT0003] Buysson RD. 1903. Note pour servir à l’histoire des Strepsiptères. Bull Soc Entomol France. 8(9):174–175.

[CIT0004] Capella-Gutierrez S, Silla-Martinez JMT. 2009. trimAl: a tool for automated alignment trimming in large-scale phylogenetic analyses. Bioinformatics. 25(15):1972–1973. 19505945 10.1093/bioinformatics/btp348 PMC2712344

[CIT0005] Carapelli A, L, Vannini, F, Nardi, JL, Boore, L, Beani, R, Dallai, F. Frati 2006. The mitochondrial genome of the entomophagous endoparasite *Xenos vesparum* (Insecta: Strepsiptera). Gene. 376(2):248–259. 16766140 10.1016/j.gene.2006.04.005

[CIT0006] Cook JL. 2014. Review of the biology of parasitic insects in the order Strepsiptera. Compar Parasitol. 81(2):134–151.

[CIT0007] Dierckxsens N, P, Mardulyn, G. Smits 2017. NOVOPlasty: de novo assembly of organelle genomes from whole genome data. Nucleic Acids Res. 45(4):e18 28204566 10.1093/nar/gkw955 PMC5389512

[CIT0008] Kathirithamby J. 2018. Biodiversity of Strepsiptera. In: Foottit RG, Adler PH, editors. Insect biodiversity: science and society. Vol II. New York: John Wiley & Sons; p. 673–703.

[CIT0009] Kathirithamby J, MS. Engel 2014. A revised key to the living and fossil families of Strepsiptera, with the description of a new family, Cretostylopidae. J Kansas Entomol Soc. 87(4):385–388.

[CIT0010] Kifune T, Maeta Y. 1985. Taxonomical studies on the genus *Xenos* (Strepsiptera, Stylopidae) parasitic on vespa and polistes (Hymenoptera, Vespidae) of Taiwan with descriptions of three new species. Japanese J Entomol. 53(3):426–435.

[CIT0011] Kumar S, G, Stecher, M, Li, C, Knyaz K. Tamura 2018. MEGA X: molecular evolutionary genetics analysis across computing platforms. Mol Biol Evol. 35(6):1547–1549. 29722887 10.1093/molbev/msy096 PMC5967553

[CIT0012] Li X, K, Ogoh, N, Ohba. X, Liang, Y. Ohmiya 2007. Mitochondrial genomes of two luminous beetles, *Rhagophthalmus lufengensis* and *R. ohbai* (Arthropoda, Insecta, Coleoptera). Gene. 392(1–2):196–205. 17300880 10.1016/j.gene.2006.12.017

[CIT0013] McMahon DP, A, Hayward, J. Kathirithamby 2009. The mitochondrial genome of the 'twisted-wing parasite' *Mengenilla australiensis* (Insecta, Strepsiptera): a comparative study. BMC Genom. 10(1):603 10.1186/1471-2164-10-603 PMC2800125 20003419

[CIT0014] Meng GL, YY, Li, CT, Yang, SL. Liu 2019. MitoZ: a toolkit for animal mitochondrial genome assembly, annotation and visualization. Nucleic Acids Res. 47(11):e63–e63. 30864657 10.1093/nar/gkz173 PMC6582343

[CIT0015] Niehuis O, G, Hartig, S, Grath, H, Pohl, J, Lehmann, H, Tafer, A, Donath, V, Krauss, C, Eisenhardt, J, Hertel, et al. 2012. Genomic and morphological evidence converge to resolve the enigma of Strepsiptera. Curr Biol. 22(14):1309–1313. 22704986 10.1016/j.cub.2012.05.018

[CIT0016] Pohl H, RG. Beutel 2008. The evolution of Strepsiptera (Hexapoda). Zoology. 111(4):318–338. 18356032 10.1016/j.zool.2007.06.008

[CIT0017] Stamatakis A. 2014. RAxML version 8: a tool for phylogenetic analysis and post-analysis of large phylogenies. Bioinformatics. 30(9):1312–1313. 24451623 10.1093/bioinformatics/btu033 PMC3998144

[CIT0018] Tang M, M, Tan, G, Meng, S, Yang, X, Su, S, Liu, W, Song, Y, Li, Q, Wu, A, Zhang, et al. 2014. Multiplex sequencing of pooled mitochondrial genomes – a crucial step toward biodiversity analysis using mito-metagenomics. Nucleic Acids Res. 42(22):e166. 25294837 10.1093/nar/gku917 PMC4267667

